# Design of a culture medium for optimal growth of the bacterium *Pseudoxanthomonas indica* H32 allowing its production as biopesticide and biofertilizer

**DOI:** 10.1186/s13568-020-01127-y

**Published:** 2020-10-23

**Authors:** Dayana Morales-Borrell, Nemecio González-Fernández, Néstor Mora-González, Carlos Pérez-Heredia, Ana Campal-Espinosa, Eddy Bover-Fuentes, Eladio Salazar-Gómez, Yissel Morales-Espinosa

**Affiliations:** 1 Department of Research and Development, Center for Genetic Engineering and Biotechnology (CIGB) of Camagüey, PO Box address: 387, 70100 Camagüey, Cuba; 2 Faculty of Forest Sciences, University of Concepcion, Concepción, Chile; 3 PROMARSA, S.A. of C.V, Guanajuato, Mexico; 4 Department of Research and Development, SynergiaBio Ltda, Maule, Chile

**Keywords:** Mixture design, Optimization, Culture media, *Pseudoxanthomonas indica*, Nematicidal activity, Bioproduct

## Abstract

Culture medium composition is one of the most important parameters to analyze in biotechnological processes with industrial purposes. The aim of this study was to design of a culture medium for optimal growth of the bacterium *Pseudoxanthomonas indica* H32 allowing its production as biopesticide and biofertilizer. The influence of several carbon and nitrogen sources and their molar ratios on *P. indica* H32 growth was investigated. The effect of different micronutrients such as mineral salts and vitamin on *P. indica* H32 growth was determined as well. A mixture design based on Design-Expert 10.0 Software was performed to optimize the culture medium concentration. Finally, in the designed medium, an attribute of the biological mechanism of action of the *P. indica* H32 against nematodes, was evaluated: the hydrogen sulfide production. It was found that tested carbon/nitrogen ratios were not a significant influence on *P. indica* H32 growth. Growth of *P. indica* H32 was favored with use of sucrose, yeast extract and phosphate buffer without the addition of any tested micronutrients. An optimal concentration of 10 g/L sucrose and 5 g/L yeast extract were obtained at a cost of 0.10 $/L. In this concentration, the specific growth rate (µ) and maximal optical density (Xmax) were equal to 0.439 h^− 1^ and 8.00 respectively. It was evidenced that under the culture conditions used, *P. indica* H32 produced hydrogen sulfide. The designed medium led to a 1.08 $/L reduction of costs in comparison to LB medium. These results were critical to carry on with biotechnological development of *P. indica* H32 as a bioproduct.

## Key points

- An optimal medium for the culture of *Pseudoxanthomonas indica* H32 was development.
- *P. indica* H32 preserve its active ingredient for nematodes control in the designed medium.

## Introduction

Plant parasitic nematodes attack almost all major crops worldwide, causing damages of over US$100 billion annually (Shah et al. 2017). Although reports about nematode-related losses in Cuba are scarce, a wide range of crops are known to have been attacked by these microorganisms, namely sugar cane, tobacco, and coffee (Díaz-Silveira and Herrera 1998). Today, the utilization of biological controls to fight plant nematodes is an attractive alternative to conventional chemical pesticides due to their persistence in the soil and proven environmentally-friendly features (Tranier et al. 2014). Previous studies, not yet published, indicate that the strain *Pseudoxanthomonas indica* H32 is the active component for a promising bioproduct candidate, with the capacity to control plant-parasitic nematodes, to control fungi, and promote plant growth (Lugo et al. 2019).

*Pseudoxanthomonas indica*, was isolated and incubated in Luria-Bertani (LB) medium by (Kumari et al. 2011). However, until now there is not a culture medium for optimal growth of the bacterium *P. indica* H32 allowing its production as biopesticide and biofertilizer. Other species of this genus have been isolated and studied, which has allowed us to know about the physiology and nutritional requirements of these bacteria (Jackson et al. 1998; Mohapatra et al. 2018; Nayaka et al. 2019). *Pseudoxanthomonas indica* does not hydrolyze urea as nitrogen source and assimilates sucrose and glucose as carbon sources (Kumari et al. 2011), so peptone and yeast extract contained in LB medium could be suitable nitrogen sources for the *P. indica* H32 growth. In addition, the LB medium contains a total mass concentration of carbon and nitrogen supplements equal to 15 g/L (10 g/L tryptone and 5 g/L yeast extract) (Protocols 2006). Therefore, this would be a criterion to consider as a starting point to design a suitable culture medium that allows optimal growth of *P. indica* H32.

The study of composition of culture medium for growth of *P. indica* H32 is critical during its biotechnological development stage, since the culture medium accounts for 30–40% of estimated production costs (Batista and Fernandes 2015). Besides, determining the nutritional, energetic, and environmental requirements of microorganisms is a critical step to develop bioprocesses (Rajendran and Thangavelu 2007). The nutritional, cellular, and biochemical environment of a bioreactor is strongly affected by the conditions of the culture and the composition of the medium, namely the carbon and nitrogen sources, mineral salts, oligoelements, peptides, amino acids, and vitamins (Sampaio et al. 2010). Culture media ensure similar nutritional conditions to the naturally existing ones, allowing for proper functioning of cell metabolism, which means an adequate balance of components (Gómez and Batista 2006).

Several methods can be used to optimize culture medium composition, including the single-factor method, though it is time-consuming, painstaking, and does not guarantee optimal condition determination (Batista and Fernandes 2015; Eswari et al. 2012; Venkata et al. 2006). Likewise, experiments based on all the possible combinations of test factors are impractical, since a large number of experiments are required (Eswari et al. 2012). One choice to increase product yields, cut down culture time and variability, as well as costs, is the application of statistical experimental design techniques (Batista and Fernandes 2015; Mu et al. 2009).

Therefore, the main objective of this study was to design a culture medium for optimal growth of the bacterium *P. indica* H32 allowing its production as biopesticide and biofertilizer, through application of statistical experimental design techniques.

## Materials and methods

### Bacterial strain

*Pseudoxanthomonas indica* H32 (Culture Collection of Microorganisms of Center of Genetic Engineering and Biotechnology (CCCIEB), Cuba, 771) stored at − 70 °C in 20% (v/v) glycerol was used in this study.

### Media screening experiments

#### Evaluation of different carbon and nitrogen sources, as well as their molar ratios on *P. indica* H32 growth

The initial composition of culture media was based on the M9 mineral medium (Geerlof 2010) (without ZnCl_2_ and CuCl_2_), which was supplemented with different carbon and nitrogen sources to study its influences on *P. indica* H32 growth. Moreover, the molar ratios of carbon (C) and nitrogen (N) sources were tested same time (C/N equal to 5, 10 and 20). Ammonium chloride (Scharlab, Spain), yeast extract (Angel, China), and bacteriological peptone z (BioCem, Cuba) were tested as nitrogen sources, whereas sucrose (TECNOAZUCAR, Cuba) and glucose (Scharlab, Spain) were evaluated as carbon sources. In the experiment, the independent variables were C, N, and the molar ratios, whereas specific growth rate (µ) and maximum optical density (Xmax) were dependent variables. The experimental design was composed by 18 point (Table 1), which were made by triplicate (n = 3). In the culture medium, the sum of the mass concentrations of the carbon and nitrogen sources studied was fixed at 15 g/L (C + N = 15), taking into consideration the composition of the LB medium (Protocols 2006).

**Table 1:** Experimental design for the evaluation of different carbon and nitrogen sources, as well as their molar ratios on *P. indica* H32 growth

Design points*	Molar ratios C/N	Carbon sources		Nitrogen sources		
		Sucrose (g/L)	Glucose (g/L)	Ammonium chloride (g/L)	Yeast extract (g/L)	Bacteriological peptone (g/L)
1	5	10.91	–	4.09	–	–
2	5	7.93	–	–	7.07	–
3	5	6.91	–	–	–	8.09
4	10	12.63	–	2.37	–	–
5	10	10.37	–	–	4.63	–
6	10	10.51	–	–	–	4.49
7	20	13.71	–	1.29	–	–
8	20	12.26	–	–	2.74	–
9	20	12.36		–	–	2.64
10	5	–	11.06	3.94	–	–
11	5	–	8.11	–	6.89	–
12	5	–	8.28	–	–	6.72
13	10	–	12.73	2.27	–	–
14	10	–	10.53	–	4.47	–
15	10	–	10.67	–	–	4.33
16	20	–	13.77	1.23	–	–
17	20	–	12.38	–	2.63	
18	20	–	12.47	–	–	2.53

* All design points was contained M9 mineral medium as basis (Geerlof 2010) without ZnCl_2_ and CuCl_2_. Moreover, the carbon and nitrogen sources corresponding to each design point was use instead of glucose (20%) present in M9 mineral medium

#### Study of the influence of mineral salts and vitamins on *P. indica* H32 growth

Seven culture media were employed to study the influence of mineral salts and vitamins present in the M9 medium on *P. indica* H32 growth. According to results of the first experiment, each culture medium contained as base 7.93 g/L sucrose, 7.07 g/L yeast extract and 100 mL/L phosphate buffer (47.8 g/L Na_2_HPO_4_ and 30 g/L KH_2_PO_4_, adjusted to pH 7.2 with NaOH and sterilized for 15 min at 121 °C). This way, the medium 1 was the base of the other six media, which were composed by **2**: medium 1 + CaCl_2_, **3**: medium 1 + MgSO_4_, **4**: medium 1 + NaCl, **5**: medium 1 + trace salts solution, **6**: medium 1 + vitamins and **7**: medium 1 + CaCl_2 +_ MgSO_4 +_ NaCl + trace salts solution + vitamins. The micronutrients were adding on equal proportion that on M9 medium (Geerlof 2010).

#### Optimization of culture medium concentration

An optimal mixture-design based on Design-Expert 10.0 Software was used to optimize culture medium concentration. The dependent variables were µ (h^− 1^), Xmax and unitary cost ($/L). The independent variables were sucrose and yeast extract concentration (0–15 g/L). The design was composed by a total of 17 experimental runs.

### Culturing conditions

From the cryopreserved culture of *P. indica* H32, 25 µL were taken and added to test tubes containing 5 mL of LB medium (previously sterilized at 121 °C during 15 min). It was incubated at 37 °C and 250 rpm on a tube rotator (Boekel Scientific Tube Spinner, USA) during 18–20 h. After this time, the OD was determined to be able to inoculate 50 mL of culture medium contained in 250 mL Erlenmeyer shake starting off initial OD equal to 0.1. Subsequently, the culture was incubated at 37 °C and 250 rpm during 52 h in a shaker incubator (New Brunswick G 25, USA). The samples were taken at 0, 4, 8, 24, 28, 32, 48, and 52 h. Bacterial growth was quantified by spectrophotometry at 530 nm (Biochrom Libra S80 spectrophotometer, UK). Absorbance was read by triplicate in each sample and OD was calculated by multiplying the absorbance value by the dilution factor. Finally, the cell growth curves were built, and µ was determined according to Raina et al. (2009).

### Assessment of the hydrogen sulfide production in the designed medium as an attribute of the biological mechanism of action of the ***P. indica*** H32 against nematodes

The method to evaluate the hydrogen sulfide production was adapted from García, Vázquez and Campos (2003) and Padilla et al. (2017):

In sterile 24-well plates (Nunc, USA), 100 µL of culture at 24 h of growing were applied by triplicate in the wells of plate destined for the samples. Then, 100 µL of 15 mmol/L cysteine (Sigma-Aldrich, USA) solution were added and mixed gently with circular movements of the plate. Subsequently, a filter paper was moistened with 10 µL of 0.1 mol/L Lead (II) acetate (Sigma-Aldrich, USA) solution in the center of each circle marked with the plate cover, to evidence the presence of the color intensity on the filter paper produced by the formation of lead sulfide. The plate was capped placing a filter paper on the cover and incubated at 37 °C during 20 h in an incubation room. After this time, the plate was carefully uncapped to stop the reaction and the results were interpreted. The culture medium was used as negative control and *Brevibacterium celere* C924, active component of the HeberNem® registered biological product for the nematode control and plant growth promoting, was used as positive control. The positive and negative controls were processed in the same way as the culture samples.

### Statistical analysis

STATGRAPHICS Centurion XVI.I was used for analysis of variance (ANOVA, α = 0.05) of all data gathered during the evaluation of the carbon and nitrogen sources tested and for study of the influence of M9 medium micronutrients on *P. indica* H32 growth. Furthermore, Software Design-Expert 10.0.1 was used for optimization of medium concentration, regression analysis, ANOVA of experimental data (α = 0.05), evaluation of goodness-of-fit, predictive model accuracy, equation of the mathematical model, R^2^ correlation coefficients, and adjusted R^2^.

## Results

### Influence of carbon and nitrogen sources and their molar ratios on *P. indica* H32 growth

**Fig. 1:**
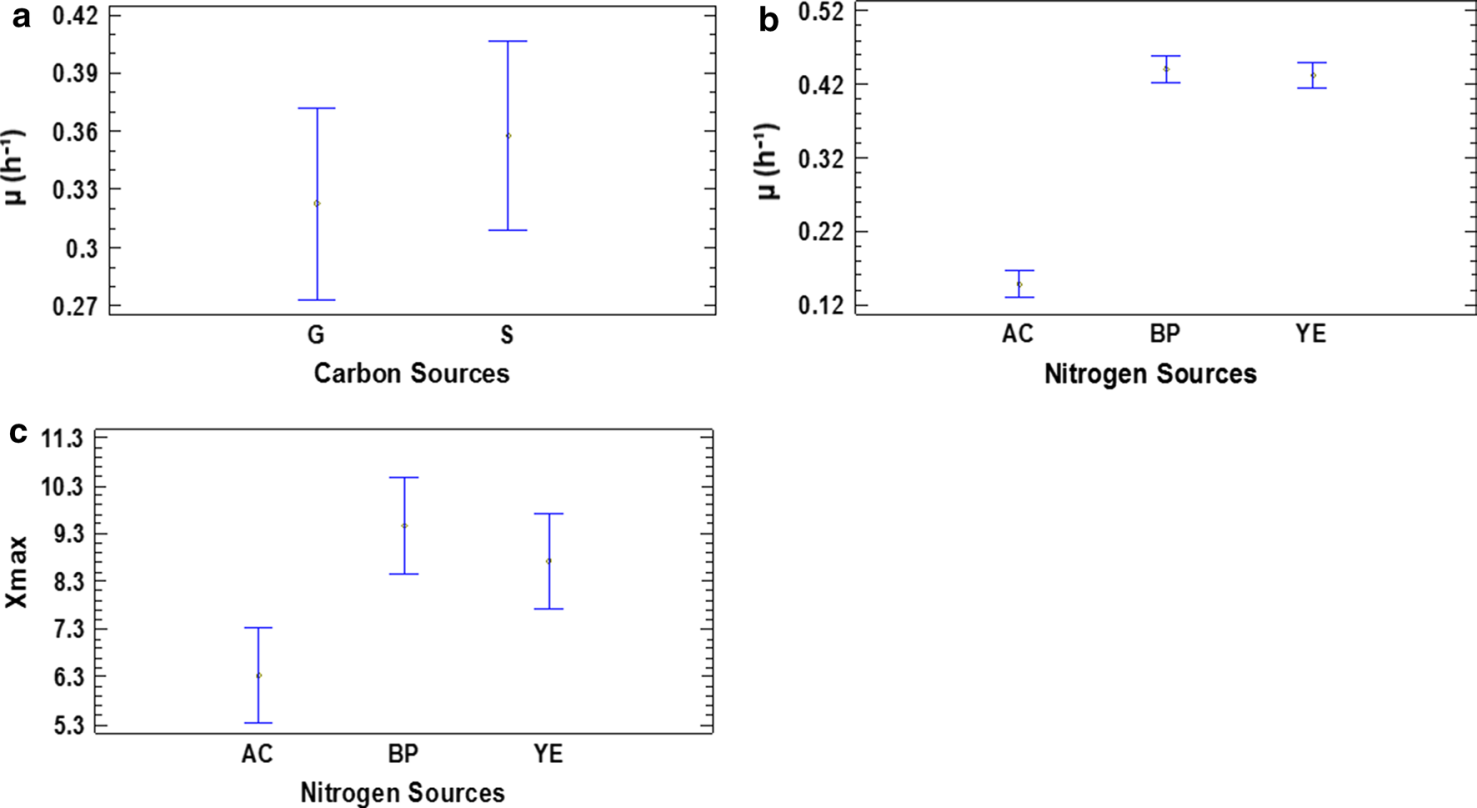
Influence of carbon and nitrogen sources on µ and Xmax of *P. indica* H32. **a** Values of µ obtained with the evaluated carbon sources. **b** Values of µ obtained with the evaluated nitrogen sources. **c** Values of Xmax obtained with the evaluated nitrogen sources. G: glucose. S: sucrose. AC: ammonium chloride. BP: bacteriological peptone. YE: yeast extract. All bars represent the mean values ± 95.0 percent LSD intervals

The carbon and nitrogen sources were selected according to the nutritional requisites of the microorganism, including availability and cost of the same. The experimental results showed that the carbon sources tested had a significant influence on µ (P = 0.0163), but not so on Xmax (P = 0.6315). With the use of sucrose and glucose as carbon sources, the µ was equal to 0.357 h^− 1^ and 0.321 h^− 1^ respectively. On the other hand, the nitrogen sources had a significant effect on µ (P = 0.0000) and Xmax (P = 0.0084). The µ was equal to 0.431 h^− 1^ and 0.441 h^− 1^ with use of yeast extract and bacteriological peptone respectively, without differences between their homogeneous groups. However, with the use of NH_4_Cl were obtained the lower values of µ (0.147 h^− 1^). The highest Xmax values were also obtained with the use of yeast extract and bacteriological peptone (9.35 and 8.58 respectively). Nevertheless, tested molar ratios were not a significant influence in the analyzed response variables (P = 0.8640 and P = 0.2192). Therefore, only factors with a significant influence (P ≤ 0.05) were analyzed through of the diagrams of Means and 95.0 Percent LSD intervals (Fig. 1). Consequently, the carbon and nitrogen sources chosen for the composition of the culture medium were sucrose and yeast extract, respectively.

### Influence of mineral salts and vitamins on *P. indica* H32 growth

**Fig. 2:**
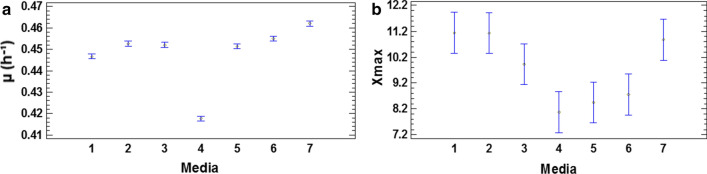
Influence of mineral salts and vitamins on *P. indica* H32 growth. **a** Values of µ obtained with the tested media. **b** Values of Xmax obtained with the tested media. **1**: medium 1 (7.73 g/L sucrose, 7.07 g/L yeast extract and 100 mL/L phosphate buffer). **2**: medium 1 + CaCl_2_. **3**: medium 1 + MgSO_4_. **4**: medium 1 + NaCl. **5**: medium 1 + trace salts solution. **6**: medium 1 + vitamins. **7**: medium 1 + CaCl_2 +_ MgSO_4 +_ NaCl + trace salts solution + vitamins. All bars represent the mean values ± 95.0 percent LSD intervals

The growth of *P. indica* H32 was influenced significantly by the composition of the culture medium. This was corroborated through ANOVA, in which the components had a significant effect on µ and Xmax, (P = 0.0000 and P = 0.0019). Each component was analyzed individually in the diagrams of Means and 95.0 Percent LSD intervals (Fig. 2). The values observed for µ were between 0.440 and 0.462 h^− 1^ except with the medium 4 (µ = 0.418 h^− 1^) (Fig. 2a). The higher Xmax values were 11.12, 11.0 and 10.93 with medium 1, medium 2 and medium 7 respectively (Fig. 2b). Medium 1 proved has only has three components (sucrose, yeast extract and phosphate buffer) allowing the simplification of the scale up fermentation process without compromising *P. indica* H32 growth, which constitutes an advantage from the technical-economic point of view. Therefore, the medium 1 was chosen to carry on with culture medium design, being named as H medium.

### Optimization of culture medium concentration

Optimization of the culture medium concentration (H medium) was made with aim to increase the specific growth rate (µ) and maximum optical density (Xmax), and to minimize the culture medium cost. The experiment results showed that with only yeast extract it was sufficient to increase the *P. indica* H32 productivity (Table 2). Nevertheless, since yeast extract was the most expensive component of the culture medium, this influenced the optimization criteria.

**Table 2:** Experimental design for optimization of culture medium concentration using I optimal mixture-design with corresponding observed values

Design points*	Components		Response 1	Response 2	Response 3
	A: Sucrose (g/L)	B: Yeast extract (g/L)	Xmax	µ (h^− 1^)	Cost ($/L)
1	15	0	0.575	0.138	0.053
2	7.5	7.5	7.320	0.433	0.123
3	5	10	7.390	0.427	0.147
4	5	10	6.960	0.433	0.147
5	7.5	7.5	7.220	0.431	0.123
6	10	5	8.000	0.439	0.100
7	10	5	7.910	0.439	0.100
8	0	15	7.170	0.452	0.194
9	3.75	11.25	5.100	0.422	0.159
10	3.75	11.25	5.680	0.395	0.159
11	11.25	3.75	5.010	0.414	0.088
12	11.25	3.75	5.310	0.421	0.088
13	7.5	7.5	6.980	0.429	0.123
14	15	0	0.580	0.156	0.053
15	15	0	0.587	0.158	0.053
16	0	15	7.280	0.451	0.194
17	0	15	6.920	0.453	0.194

* All design points was contained 100 mL/L phosphate buffer (47.8 g/L Na_2_HPO4 and 30 g/L KH2PO_4_)

Thus, the Xmax values raised were more adjusted to a quartic mixture model (R^2^ = 95.32%, and adjusted R^2^ = 93.76%). Factors A, B, AB, AB (A-B), AB (A-B)^2^ were considered significant terms of the model (P < 0.05) (Table 3). The mathematical model equation obtained for Xmax was:

**Table 3:** ANOVA for quartic mixture model for the response 1: Xmax

Mixture component coding is L_Pseudo						
Analysis of variance table [Partial sum of squares—Type III]						
Source	Sum of square	df	Mean square	F Value	p-value Prob > F	
Model	101.90	4	25.47	61.14	< 0.0001	Significant
Linear mixture	50.23	1	50.23	120.56	< 0.0001	
AB	34.98	1	34.98	83.95	< 0.0001	
AB(A-B)	14.45	1	14.45	34.67	< 0.0001	
AB(A-B)2	2.41	1	2.41	5.78	0.0333	
Residual	5.00	12	0.42			
Lack of fit	4.56	2	2.28	51.96	< 0.0001	Significant
Pure error	0.44	10	0.044			
Cor total	106.90	16				

$$Xmax = 0.037486A+0.474604B+0.068911AB+0.005245AB\left(A-B\right)-0.000437AB{\left(A-B\right)}^{2}$$

Likewise, the µ values achieved, fit a cubic mixture model better with R^2^ = 99.21%, and adjusted R^2^ = 99.02%. Factors A, B, AB, AB (A-B), were considered significant terms of the model (Table 4). The mathematical model equation obtained for ${\upmu }$ was:

**Table 4:** ANOVA for Cubic Mixture Model for the response 2: µ

Mixture component coding is L_Pseudo						
Analysis of variance table [Partial sum of squares—Type III]						
Source	Sum of squares	df	Mean square	F value	p-value Prob > F	
Model	0.20	3	0.066	541.12	< 0.0001	Significant
Linear mixture	0.11	1	0.11	877.10	< 0.0001	
AB	0.060	1	0.060	497.72	< 0.0001	
AB(A-B)	0.030	1	0.030	248.55	< 0.0001	
Residual	1.576E−003	13	1.212E−004			
Lack of fit	9.114E−004	3	3.038E−004	4.57	0.0290	Significant
Pure error	6.646E-004	10	6.646E−005			
Cor total	0.20	16				

$${\upmu } = 0.010134A+0.030214B+0.002505AB+0.000240AB\left(A-B\right)$$

The ANOVA of cost variable, manifest that only factors A and B were significant terms of the model since (Table 5). The cost values obtained, fit a linear mixture order model, with R^2^ = 100% and adjusted R^2^ = 100%. The mathematical model equation obtained for cost was:

**Table 5:** ANOVA for linear mixture model for the response 3: cost

Mixture component coding is L_Pseudo						
Analysis of variance table [Partial sum of squares—Type III]						
Source	Sum of squares	df	Mean square	F value	p-value Prob > F	
Model	0.037	1	0.037	6.366E + 007	< 0.0001	Significant
Linear mixture	0.037	1	0.037	6.366E + 007	< 0.0001	
Residual	0.000	15	0.000			
Lack of fit	0.000	5	0.000			
Pure error	0.000	10	0.000			
Cor total	0.037	16				

$$Cost = 0.003555A+0.012905B$$

**Fig. 3:**
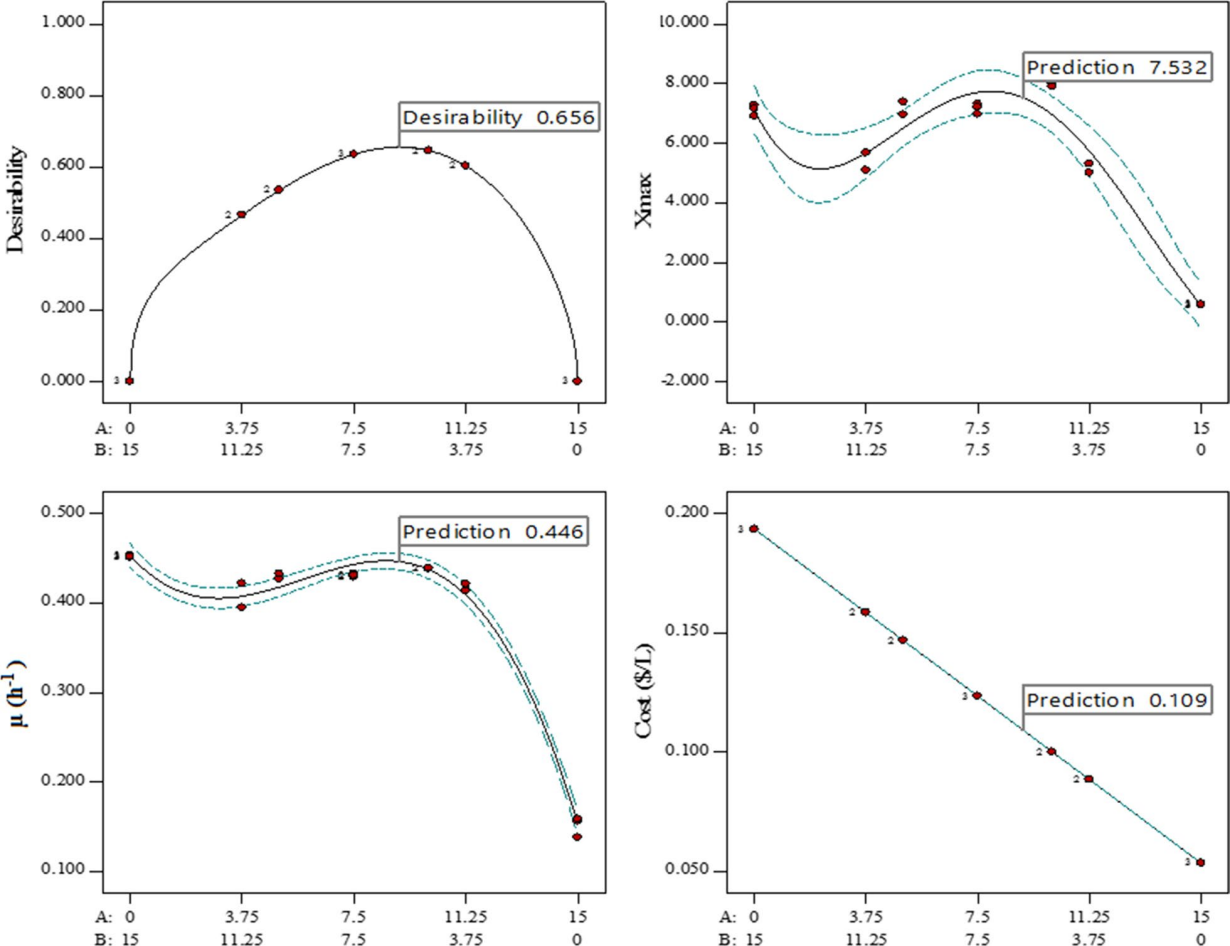
Predicted optimum of the response variables from the optimization of the culture medium (H medium) concentration using the two-component mixture. A: Sucrose. B: Yeast extract. All experimental points of the design contain the same proportion of phosphate buffer (100 mL/L).  Design points

The solution of the optimization for the H medium concentration had a desirability of 0.656. The optimal concentration predicted was 9.28 g/L of sucrose and 5.72 g/L of yeast extract, respectively (Fig. 3). For validation, 10 g/L of sucrose and 5 g/L of yeast extract were used. The observed value was between the percentage of interval (PI) predicted with 95% confidence (Table 6).

**Table 6:** Validation of optimal concentration predicted

Response	Prediction	Std dev	SE (n = 3)	95% PI low	Observed mean	95% PI high
Xmax	7.532	0.645	0.467	6.398	8.00	8.435
µ (h^− 1^)	0.446	0.011	0.008	0.428	0.439	0.461
Cost ($/L)	0.109	0	0	0.107	0.100	0.107

The H medium is 11.84-fold less costly than the LB medium (Table 7). Consistently, similar and higher values of µ and Xmax than LB medium were achieved, which demonstrated the suitability of the culture medium designed for the *P. indica* H32 growth and scale up fermentation process.

**Table 7:** Ratio between the optimized medium (H medium) and LB medium

Parameters	LB medium	H medium	LB/ H ratio
Xmax	8.30	8.00	1.037
µ (h^− 1^)	0.39	0.44	0.886
Cost ($/L)	1.18	0.10	11.840

### Hydrogen sulfide production in the designed medium as an attribute of the biological mechanism of action of the ***P. indica*** H32 against nematodes

**Fig. 4:**
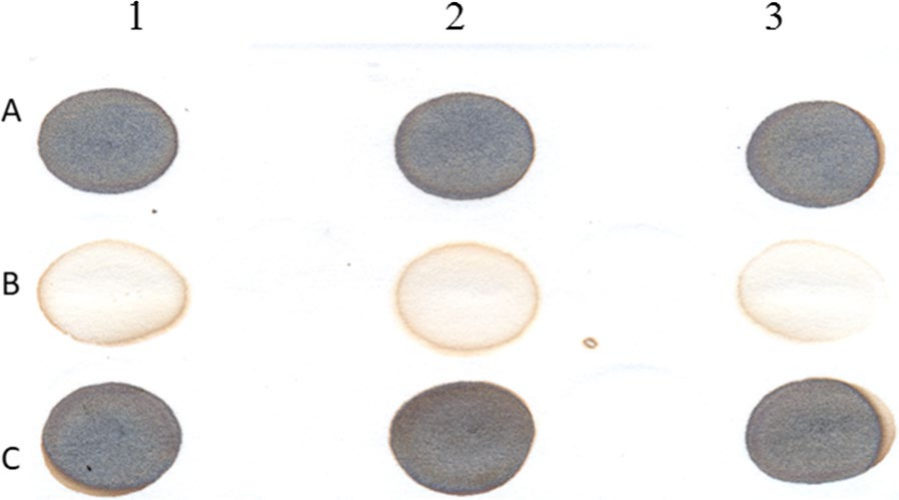
Hydrogen sulfide production by the *P. indica* H32 at 24 h of culture in the H medium. 1, 2 and 3 number of replicas. A: *P. indica* H32. B: Negative control (H medium). C: Positive control (*Brevibacterium celere* C924, active component of the HeberNem® registered biological product for the nematode control and plant growth promoting)

As a result of this experiment, the production of hydrogen sulfide by the bacterium *P. indica* H32 was evidenced, through the qualitative determination of the dark spots on the filter paper generated by lead sulfide. The intensity of the spots on the filter paper corresponding to the production of hydrogen sulfide by *P. indica* H32 were higher than the samples of the negative control (H medium) and similar to the samples of the positive control (*Brevibacterium celere* C924) (Fig. 4).

## Discussion

Process optimization is an essential procedure in biotechnology industry to guarantee higher yield with a minor cost. The design and optimization of medium nutritional parameters such as composition and nutrients ratios, is rather effective to improve fermentation process. Therefore, the use of cheap and available sources of nitrogen and carbon is desirable for the optimization of fermentation as this can significantly reduce the cost of production. This study was conducted to develop a culture medium for optimal growth of the bacterium *P. indica* H32 allowing its production as biopesticide and biofertilizer.

The influence of different carbon and nitrogen sources on *P. indica* H32 growth was study. Regarding the effect of organic nitrogen sources, yeast extract and bacteriological peptone, exhibited the most increase of µ. The effect exerted by ammonium chloride on µ was negligible in comparison with yeast extract and bacteriological peptone. The unlike on µ according the use of different nitrogen sources can be explained by their chemical composition. Yeast extract and bacteriological peptone have amino acids and protein, among other micronutrients (Podpora et al. 2016), which are essential for growth, whereas ammonium chloride mostly provide nitrogen.

Meanwhile, the evaluated carbon sources also had significant influence on µ of *P. indica* H32, although the use of sucrose as carbon source allowed reaching a value of µ of 1.11 times higher than using glucose. Other studies about of growth requirements for production of the bioherbicidal by bacterium *Xanthomonas campestristhe* (phylogenetically related genus with the *P. indica* H32) show that the substitution of sucrose with glucose did not significantly alter cell densities (Jackson et al. 1998). Many bacteria possess catalytic machinery to metabolize sucrose and the genes comprising sucrose catabolic operons are strictly regulated (Saier et al. 1995). The fact that the higher value of µ was achieved with sucrose (being glucose in most of cases a preferential carbon source), suggests that *P. indica* H32 possess a functional and regulable system for sucrose uptake and catabolism, capable of using sucrose efficiently when it is present.

The evaluation of mineral salts and vitamins on *P. indica* H32 growth demonstrate that only with carbon, nitrogen and phosphate buffer supplementation in the culture medium (medium 1, named as H medium) is sufficient to increase *P. indica* H32 growth, without addition of any micronutrient. Similar results were found by Jackson et al. (1998), who showed that omission of the vitamin mixture in the culture medium did not significantly alter cell densities. They report that the presence of sucrose or glucose as the carbon source and various organic nitrogen sources in the culture medium, supported optimal *X. campestris* growth and cell yield.

The study of optimization of culture medium concentration, allowed obtaining high values of Xmax and µ of the bacterium *P. indica* H32 with a lower expenditure of sucrose and yeast extract (Fig. 4). This led to a reduction in the cost of the culture medium, which is essential for processes with industrial purposes.

Also, the hydrogen sulfide production by *Brevibacterium celere* C924, has been associated with its biological mechanism of action against nematodes (García et al. 2003; Padilla et al. 2017). Therefore, this attribute was considered as a criterion for the assessment of the biological mechanism of action of the *P. indica* H32 against nematodes. The results of this experiment were consistent with those obtained by (Padilla et al. 2017). These findings demonstrate that in the designed medium, *P. indica* H32 not only can grow satisfactorily, also support its active principle, which is the main characteristic that must remain.

In conclusion, a new culture medium (H medium) was designed for the optimal growth of the bacterium *P. indica* H32 allowing its production as biopesticide and biofertilizer. Optimal concentration of the H medium was 10 g/L of sucrose and 5 g/L of yeast extract with 100 mL/L of phosphate buffer in the medium. In this concentration, the values achieved of µ and Xmax were equal to 0.439 h^− 1^ and 8.00 respectively. H medium has a simple composition, allowing the simplification of the scale up fermentation process, without compromising the *P. indica* H32 growth and its biological mechanism of action against nematodes. It also has considerably lower cost than the LB medium and at the same time, similar and higher values of Xmax and µ are reached. This finding demonstrated the suitability of the culture medium designed for the growth and scale up fermentation of *P. indica* H32. Nevertheless, further studies are required to optimize the fermentation conditions of the *P. indica* H32.

## Data Availability

The data that support the findings of this study are available from Center for Genetic Engineering and Biotechnology (CIGB) of Camagüey but restrictions apply to the availability of these data, which were used under license for the current study, and so are not publicly available. Data are however available from the authors upon reasonable request and with permission of Center for Genetic Engineering and Biotechnology (CIGB) of Camagüey.
